# Diabetes Alert Day — March 26, 2013

**Published:** 2013-03-22

March 26 is Diabetes Alert Day, which is dedicated to raising awareness about type 2 diabetes, its risk factors, and its prevention. Type 2 diabetes, which can be prevented or delayed through lifestyle changes such as losing weight and increasing physical activity, accounts for 90%–95% of all diabetes cases in the United States (1).

Information about type 2 diabetes and ways to prevent it is available from numerous sources. The Prediabetes Risk Test (http://www.cdc.gov/diabetes/prevention/prediabetes.htm) is a helpful resource that uses answers to a few simple questions about weight, age, family history, and other risk factors to indicate a person’s risk for developing type 2 diabetes.

The CDC-led National Diabetes Prevention Program (http://www.cdc.gov/diabetes/prevention/index.htm) is working with partners in communities across the United States to establish effective lifestyle change programs for persons at high risk for type 2 diabetes. Lifestyle change programs are listed by state at http://www.cdc.gov/diabetes/prevention/recognition/registry.htm#program. The Just One Step tool (http://ndep.nih.gov/resources/diabetes-healthsense/just-one-step.aspx), created by the National Diabetes Education Program, a joint program of the CDC and the National Institutes of Health, provides helpful tips for making lifestyle changes.

CDC’s Diabetes Interactive Atlases (http://www.cdc.gov/diabetes/atlas) provide data on trends in diagnosed diabetes (both prevalence and incidence), obesity, and leisure-time physical inactivity in the United States. Additional information about diabetes control and prevention is available at http://www.cdc.gov/diabetes.
